# Silent Signals: Unveiling Cochlear Nerve Deficiency in a Child Through Imaging

**DOI:** 10.7759/cureus.72582

**Published:** 2024-10-28

**Authors:** Sanjay M Khaladkar, Ojasvi Sharma, Saksham Jain, Akhil Varma

**Affiliations:** 1 Radiodiagnosis, Dr. D.Y. Patil Medical College, Hospital and Research Centre, Dr. D.Y. Patil Vidyapeeth (Deemed to be University), Pune, IND

**Keywords:** cochlear nerve, congenital sensorineural hearing loss, hrct temporal bone, internal auditory canal, temporal bone mri, vestibulocochlear nerve

## Abstract

Cochlear nerve deficiency (CND) is a rare cause of congenital sensorineural hearing loss, often leading to serious communication challenges if not diagnosed and treated early. We present this case of a three-year-old male child with congenital hearing loss and sensory aphasia. Despite normal cochlear anatomy on computed tomography, magnetic resonance imaging (MRI) revealed the absence of the cochlear nerve on the right side. The diagnosis of right-sided CND was confirmed, and the patient was advised to undergo cochlear implantation and speech therapy. This case underscores the importance of timely imaging, particularly using MRI with high-resolution sequences, in diagnosing CND and facilitating early intervention to improve auditory and developmental outcomes.

## Introduction

Approximately 20% of patients with congenital sensorineural hearing loss (SNHL) exhibit radiographic anomalies in the inner ear. A wide range of pathologies can be responsible for these abnormalities [1]. The terms cochlear nerve deficiency (CND) or cochlear nerve (CN) hypoplasia or aplasia refer to a condition where the CN is either underdeveloped or completely absent. Based on radiological findings, these malformations have been estimated to be present in 0.8% to 1.8% of profoundly deaf children.

Magnetic resonance imaging (MRI) is required to assess whether the nerve is defective, though it is often difficult to differentiate between hypoplasia and aplasia due to resolution limitations, especially when the internal auditory canal (IAC) is narrow [2]. However, a missing or dysfunctional cochlear branch of the vestibulocochlear nerve (VCN) could be the reason for congenital deafness and can be assessed with sub-millimetric T2-weighted gradient echo imaging. This imaging technique enables clear visualization of various cranial nerves and their branches within the internal auditory canal and cerebellopontine angle. Another effective method for identifying nerves within the IAC is axial three-dimensional Fourier transformation-constructive interference in steady state (3DFT-CISS) sequence, which facilitates identification of the cochlear branch, inferior vestibular branch, and superior vestibular branch of the VCN [1,3].

However, even if the MRI reveals CN aplasia, a full battery of audiometric tests needs to be conducted to rule out other etiological factors before arriving at the final diagnosis.

Here, we describe the case of one such child, highlighting the role of radiological investigation in cementing the diagnosis.

## Case presentation

This case is of a three-year-old male child who presented to the pediatric outpatient department with congenital hearing loss and sensory aphasia. The mother first noticed the condition when the child was six months old as he did not respond to loud sounds, particularly on the right side. As the child grew older, he failed to respond to his name or follow commands, and his parents observed that he was unable to vocalize words like other children in his age group.

The child was his parents' firstborn and was delivered at term via lower segment cesarean section (LSCS) due to being large for gestational age (LGA). He cried immediately after birth, and there was no requirement for a Neonatal Intensive Care Unit (NICU) admission for any perinatal complications. His immunizations were up to date according to the recommended schedule. The mother had not observed any delays in his motor or fine sensory development.

Family history revealed that the parents’ marriage was non-consanguineous. The mother also suffers from congenital hearing loss and aphasia but did not undergo any investigations to identify the cause for the same. The father, though unable to speak since childhood, has normal hearing. Although syndromes like Usher, Wolfram, and Pendred syndrome are associated with congenital hearing loss, in this case, the child presented solely with bilateral SNHL with sensory aphasia and no other abnormalities, indicating a likely non-syndromic etiology.

For evaluating the child’s hearing loss, a Brainstem Evoked Response Audiometry (BERA) was performed at another hospital and it indicated bilateral hearing loss (profound on the right side, severe on the left side).

**Figure 1:**
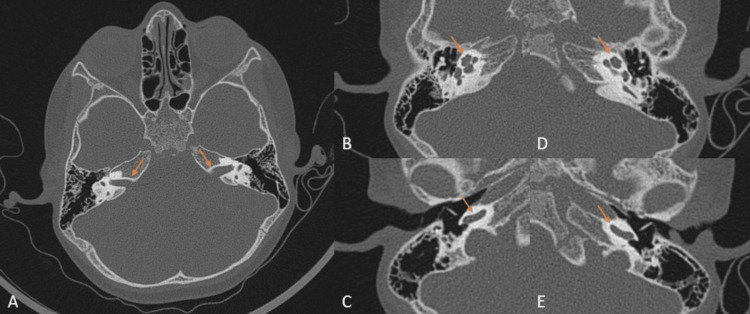
High-resolution computerized tomography of the temporal bone A – Bilateral internal auditory canals appear normal (arrows). B – Right cochlear apical and middle turn appear normal (arrow). C – Right cochlear basal turn appears normal (arrow). D – Left cochlear apical and middle turn appear normal (arrow). E – Left cochlear basal turn appears normal (arrow).

High-resolution computed tomography (HRCT) of the temporal bone was done in November 2023 using the Philips Ingenuity 128-slice CT scanner (Philips Healthcare, Amsterdam, Netherlands). It revealed a normal cochlea with no structural abnormalities of the inner ear (Figures 1A-1E).

**Figure 2:**
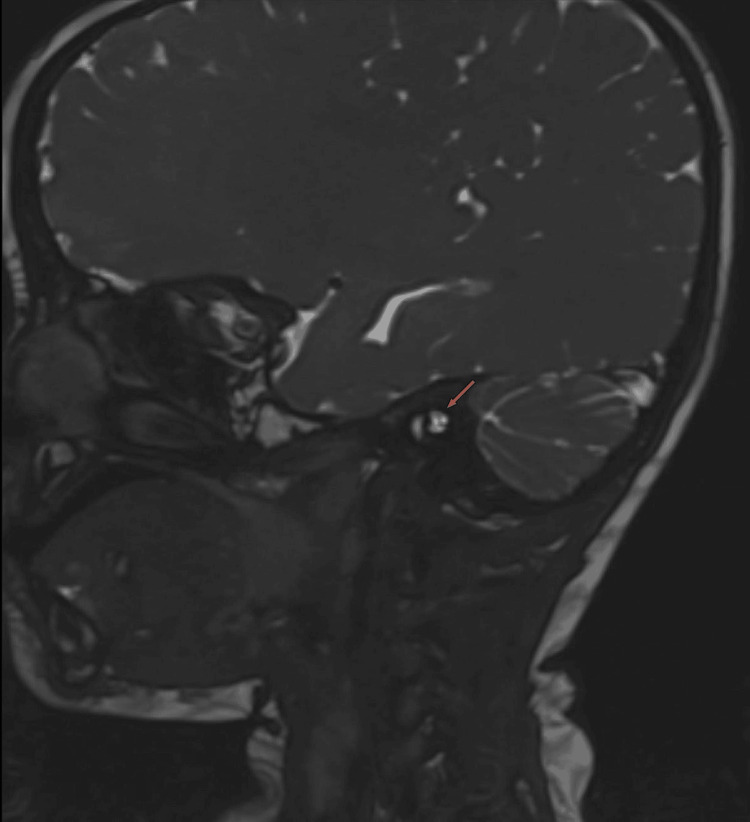
Magnetic resonance image of the right temporal bone Facial nerve and vestibular nerves seen within the right internal auditory canal (arrow) appear normal. However, cochlear division of the vestibulocochlear nerve is not visualized in the antero-inferior quadrant.

Plain MRI of the temporal bone was done in February 2024 using the Siemens 3 Tesla MAGNETOM Vida MRI (Siemens Healthineers, Erlangen, Germany) with thin T1-weighted images and thin T2-weighted images in axial and coronal planes and a Constructive Interference in Steady State (CISS) sequence. On the right side, the cochlear division of the VCN was not seen in the antero-inferior quadrant, suggestive of CND (either marked hypoplasia or agenesis) (Figure 2).

**Figure 3:**
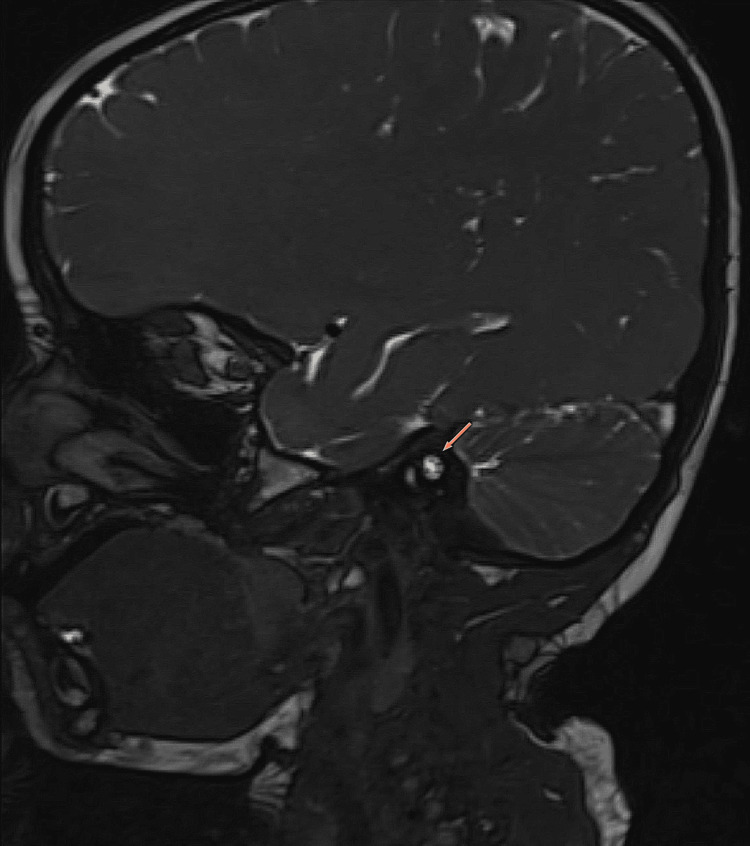
Magnetic resonance image of the left temporal bone Normal appearance of the cochlear nerve seen within the internal auditory canal (arrow) in the antero-inferior quadrant with facial nerve seen in the antero-superior quadrant. Superior and inferior vestibular nerves are also visualized in the postero-superior and postero-inferior quadrants respectively.

However, on the left side, the internal auditory canal showed normal appearance of the cochlear division of VCN in the antero-inferior quadrant (with a calibre larger than the adjacent facial nerve) (Figure 3).

**Figure 4:**
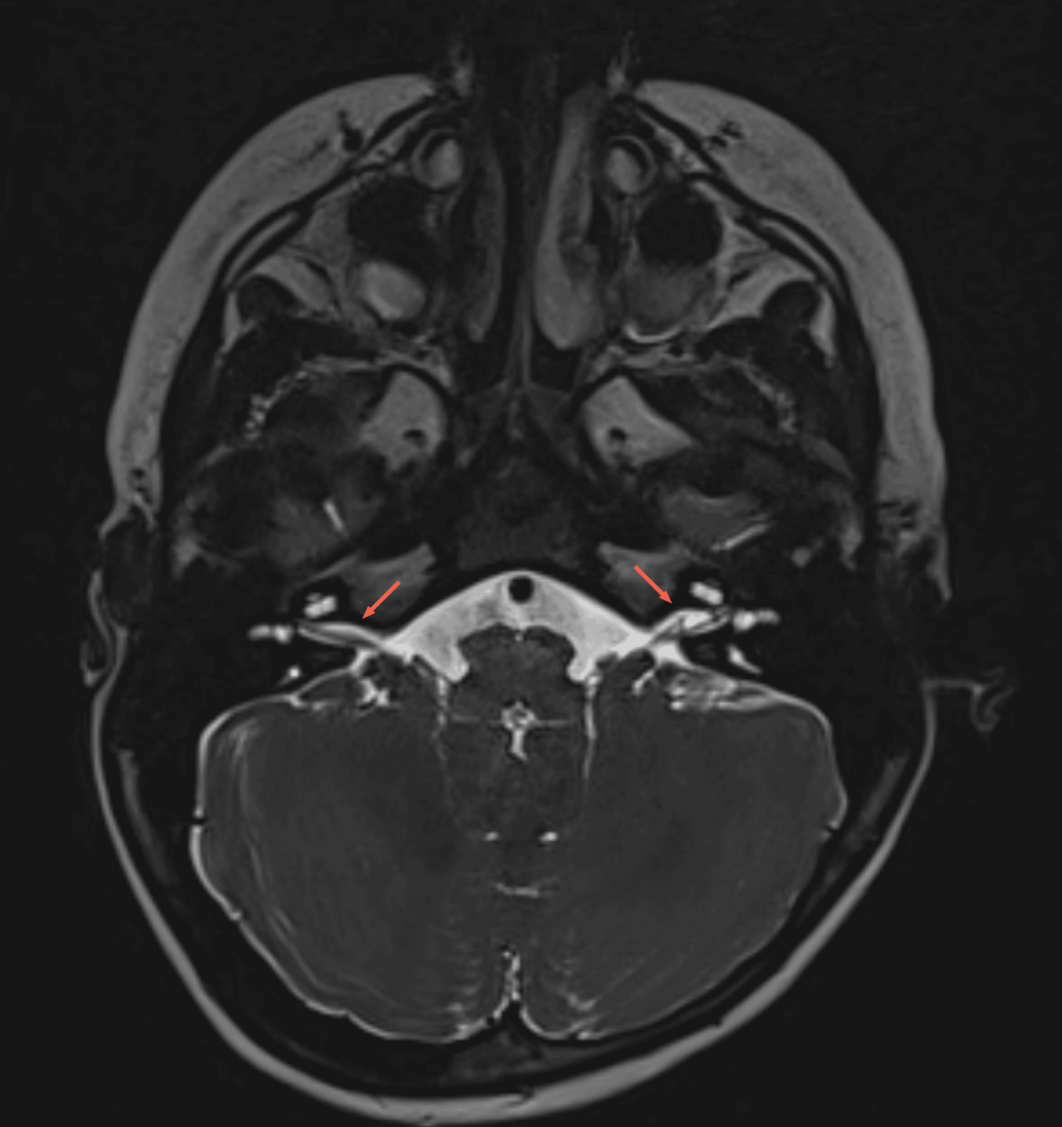
Magnetic resonance image with the bilateral internal auditory canals appearing normal (arrows)

Bilateral internal auditory canals were normal (Figure 4).

Based on this, a diagnosis of right CND was made. The parents were counselled regarding cochlear implants followed by appropriate speech therapy.

## Discussion

An MRI of the temporal bone is a crucial diagnostic tool for cases of congenital profound sensorineural deafness, as it is the only test capable of evaluating the structure of the VCN and its branches [4,5].

High resolution and excellent soft tissue/fluid contrast are offered by the standard IAC protocol that uses T2-weighted gradient echo or turbo gradient-spin-echo sequences with sub-millimeter inter-slice spacing and slice thickness [6]. In the present case report, CISS imaging was crucial to confirm the absence of the cochlear nerve on the right side. Additionally, imaging can be done in the axial plane, using either direct acquisition or multi-planar reconstruction to obtain bilateral parasagittal images perpendicular to the nerves. While the latter results in longer scan periods, it offers higher resolution [7].

In a study done by Clemmens et al. in 2013, 128 children with unilateral SNHL underwent a high-resolution MRI study [8]. The CN's diameter, area, and signal intensity were assessed and compared to the ipsilateral facial nerve. CND was found in 26% of children with unilateral SNHL, with a higher occurrence in those with profound SNHL.

In a study conducted by Nakano et al., 170 children with congenital SNHL were evaluated using CT scans [9]. CND was defined as stenosis of the bony cochlear nerve canal (BCNC), IAC, or both. CND was detected in 50% of the cases with unilateral SNHL compared to 5.3% of cases with bilateral SNHL. The majority of the CND cases were associated with syndromes or cochlear malformations. However, the cochlear anatomy was normal in the present case and unilateral CN agenesis was verified on the MRI scan.

While we couldn’t delineate any genetic syndrome associated with the unilateral SNHL in this case, the possibility of additional symptoms and signs emerging over time remains. Therefore, regular screening and follow-up are essential. In their review, Peng et al. observed that better auditory outcomes in these patients are associated with both the presence of a cochlear nerve on the MRI study and the absence of concomitant medical syndromes [10].

## Conclusions

Cochlear nerve agenesis or hypoplasia is a rare condition and a known cause of SNHL, more frequently associated with unilateral than bilateral SNHL. Although it has been studied extensively, diagnosing this condition can be challenging due to the difficulties associated with imaging of the narrow IAC, which is often associated with CND. Hence, in addition to audiometric tests and HRCT of the temporal bone, an MRI is also essential to screen patients presenting with congenital SNHL to evaluate the CN for any abnormalities. Furthermore, for patients with a family history of congenital deafness, early screening is important as it can enhance auditory outcomes and mitigate the risk of subsequent developmental anomalies like aphasia.
